# On Quality Control Measures in Genome-Wide Association Studies: A Test to Assess the Genotyping Quality of Individual Probands in Family-Based Association Studies and an Application to the HapMap Data

**DOI:** 10.1371/journal.pgen.1000572

**Published:** 2009-07-24

**Authors:** David W. Fardo, Iuliana Ionita-Laza, Christoph Lange

**Affiliations:** 1 Department of Biostatistics, University of Kentucky College of Public Health, Lexington, Kentucky, United States of America; 2 Department of Biostatistics, Harvard School of Public Health, Boston, Massachusetts, United States of America; 3 Channing Laboratory, Brigham and Women's Hospital, Harvard Medical School, Boston, Massachusetts, United States of America; University of Geneva Medical School, Switzerland

## Abstract

Allele transmissions in pedigrees provide a natural way of evaluating the genotyping quality of a particular proband in a family-based, genome-wide association study. We propose a transmission test that is based on this feature and that can be used for quality control filtering of genome-wide genotype data for individual probands. The test has one degree of freedom and assesses the average genotyping error rate of the genotyped SNPs for a particular proband. As we show in simulation studies, the test is sufficiently powerful to identify probands with an unreliable genotyping quality that cannot be detected with standard quality control filters. This feature of the test is further exemplified by an application to the third release of the HapMap data. The test is ideally suited as the final layer of quality control filters in the cleaning process of genome-wide association studies. It identifies probands with insufficient genotyping quality that were not removed by standard quality control filtering.

## Author Summary

Genome-wide association studies have led to the discovery of many novel, reproducible associations between genetic loci and disease phenotypes. An important step in the analysis of genome-wide association studies is the data cleaning/QC filtering step. The statistical analysis tools that are applied as QC filters typically include testing for Hardy-Weinberg equilibrium, testing for Mendelian inconsistencies, evaluating quality scores, etc. We propose a new genome-wide transmission test for family-based designs that is applied to the dataset after the QC filtering. It allows for the assessment of the genotyping error rate that is caused by miscalled genotypes that could not be detected by the QC filters. By applying the test to individual probands, probands with insufficient genotyping quality can be identified and removed from the dataset before the analysis.

## Introduction

Over the last several years, genome-wide association studies (GWAS) have led to the identification of numerous, replicable associations between novel genetic loci and complex diseases/phenotypes [1]–[13]. While the technological breakthroughs in genotyping technology provide a wealth of information and an unbiased look at almost the entire human genome [14]–[16], the statistical analysis of such studies is not trivial and the development of new analysis methods is still ongoing. Besides the inherent multiple testing problem in such studies, the genotype data processing and cleaning steps present a statistical challenge, even before the genetic association analysis can take place. In the data cleaning step, basic statistical analysis tools are utilized as quality control measures/filters to identify markers and probands for whom the genotypic quality is problematic [17]. By filtering out markers and probands with insufficient genotype quality, the subsequent association analysis can be focused on the subset of reliable markers and probands. The overall statistical power of the study will thereby be increased and the number of false positive findings will be reduced. The statistical analysis tools that are applied in the quality control filtering step typically include testing for Hardy-Weinberg equilibrium, testing for Mendelian inconsistencies, evaluating quality scores, etc.

However, even after the most careful quality control filtering, one has to recognize that it will not be possible to detect all inherent genotyping errors in the dataset and eliminate their influence on an association analysis. For many of the SNPs and probands, the genotyping error rate will not be “poor” enough to raise a “red flag” in the quality control filtering step and it will not be possible to remove them from the dataset for the association analysis.

Here we propose a new quality control filter for family-based studies that allows the researcher to assess the genotyping quality of each proband by looking at the transmission patterns of the minor and the major allele within the same proband. That is, the new filter provides an additional evaluation of data quality at the proband level. For example, in a nuclear family in which one proband and both of the proband's parents have been genotyped, we can compare the number of SNPs for which the minor allele is transmitted from the heterozygous parents to the number of SNPs for which the major allele is transmitted. Since the null hypothesis of no association will be true for the vast majority of SNPs, we expect to observe about the same number of minor allele transmissions as major allele transmissions. However, in the presence of systematic genotypic error, this will be different. Undetected genotyping error can create larger numbers of transmissions of the major allele than the minor allele [18],[19]. In contrast to standard family-based tests [20] that examine the transmission pattern for all study participants at a specific genetic locus, we propose here a transmission test that assesses the transmission patterns at a genome-wide level for a single proband in a nuclear family. Consequently, the test can be used to measure the genotyping error rate of each proband individually. In simulation studies, we show that, for sample sizes, error rates and allele frequencies often observed in practice, the proposed test is sufficiently powerful to identify probands with unreliable genotyping quality that were not detected by standard quality control filters. An application to the third release of the HapMap data illustrates this important feature of the test further.

## Methods

In the presence of genotyping error, it is a well-understood phenomenon that standard family-based association tests (Transmission Disequilibrium Test [20], Family-Based Association Tests [21], etc) are biased under the null hypothesis and do not maintain the pre-specified *α*-level [19],[22],[23]. Under standard genotyping error models, more transmissions of the common allele will be observed than can be expected just by chance under the assumption of Mendelian transmissions [18],[19]. In a genome-wide association study, after the quality control filtering of all genotyped SNPs, the genotyping error rate for each individual SNP is expected to be small and departures of the transmission pattern from the null hypothesis that are caused by genotyping errors are unlikely detectable by a single locus analysis.

In order to estimate the undetected genotyping error for an individual proband after quality control filtering, the information about the transmission patterns has to be aggregated across all of the proband's genotyped SNPs. Consequently, we define for each proband an individual transmission test statistic that can be used to infer the underlying, undetected average genotyping error rate for the selected proband.

In order to keep the notation simple, we assume that one trio is available for genotyping at 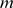 bi-allelic marker loci. The variable 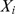 denotes the number of target/minor alleles in the proband of the trio at the 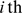 marker locus based on a called genotype. It, therefore, reflects any errors in genotyping and is not necessarily equal to the true allele totals. Similarly, the parental counts at the 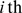 locus are given by 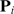. Then for 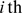 marker locus, we can define the Mendelian residual by
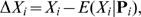
(1)where 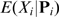 is computed based on the assumption of Mendelian transmissions. When the parental genotypes are unknown and genotypic information on additional probands is available, the parental genotypes in equation (1) can be replaced by the sufficient statistic of Rabinowitz & Laird [24]. Based on the Mendelian residuals, a genome-wide transmission score for the proband in the trio can be constructed as
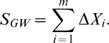
(2)


By summing over the Mendelian residuals 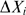 for all genotyped markers in the proband, the score 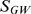 assesses the Mendelian transmission patterns globally and evaluates the null hypothesis of no preferential transmission of the minor allele at a genome-wide level. Given the SNP density on the currently used SNP chips, some proportion of the SNPs will be in linkage disequilibrium (LD). The potential correlation between the SNPs has to be taken into account when the variance of 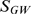 is computed in order to standardize the test statistic. Standard approaches for the computation of the variance, as they are used, for example in the TDT or FBAT statistic, assume independence of the Mendelian residuals 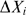 and are therefore not applicable here.

However, the asymptotic properties of 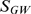 can be derived without knowledge of the LD structure by interpreting 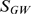 as a permutation test statistic. For the computation of the Mendelian residual at each SNP, an allele has to be selected as the target allele. For a bi-allelic marker locus, an exchange of the target allele implies a change in the sign of the Mendelian residual, i.e. 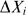 changes to 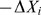. Under the null hypothesis of no preferential transmission of either allele at a genome-wide level, the assignment of the target allele at each SNP can be considered as a random selection process, with selection probability 50% for each allele and with independent draws at each SNP locus. The absence or presence of LD between the SNPs does not affect the validity of this permutation argument, since the Mendelian residuals are treated here as fixed and the sign of the residual is selected randomly with equal probabilities. Hence, under the null hypothesis of no preferential transmission, the expected value of 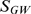 and its variance are given by 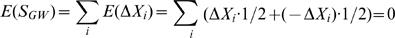 and 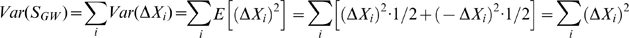 for any user-specified choice of target alleles at the genetic loci under consideration. Although derived in a different context, this variance estimator is similar to the empirical variance estimator that is used in the pedigree disequilibrium test [25]. Here, under the null hypothesis of no preferential transmission of one allele, the standardized genome-wide transmission statistic, 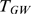, is
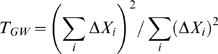
(3)and will have an approximate 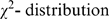 with 1 df when the null hypothesis (of no genotyping errors) is true. In our application of the genome-wide transmission statistic 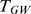, we will select the minor allele as the target allele for all SNPs. In the presence of genotyping errors across SNPs, the minor allele is expected to be under-transmitted, i.e. more negative Mendelian residuals than just by chance are expected [18],[19]. Consequently, by selecting the minor allele as target allele for all SNPs in the specification of 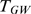, we obtain a test statistic that will assess genotype error across all SNPs within one proband.

Since the sample size for the genome-wide transmission statistic 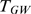 is the number of statistically independent SNPs on a particular chip, the proposed test will have a sample size of at least tens of thousands for most commercially available SNP chips. Consequently, for sample sizes, error rates and allele frequencies often observed in practice, the genome-wide transmission statistic 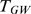 will have sufficient power to detect small to moderate departures from the Mendelian transmission patterns that are caused by genotyping errors, even though 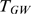 is computed for only one proband. This theoretical property is verified and quantified in subsequent simulation studies.

## Results

### Simulation Studies—Power

**Figure 1:**
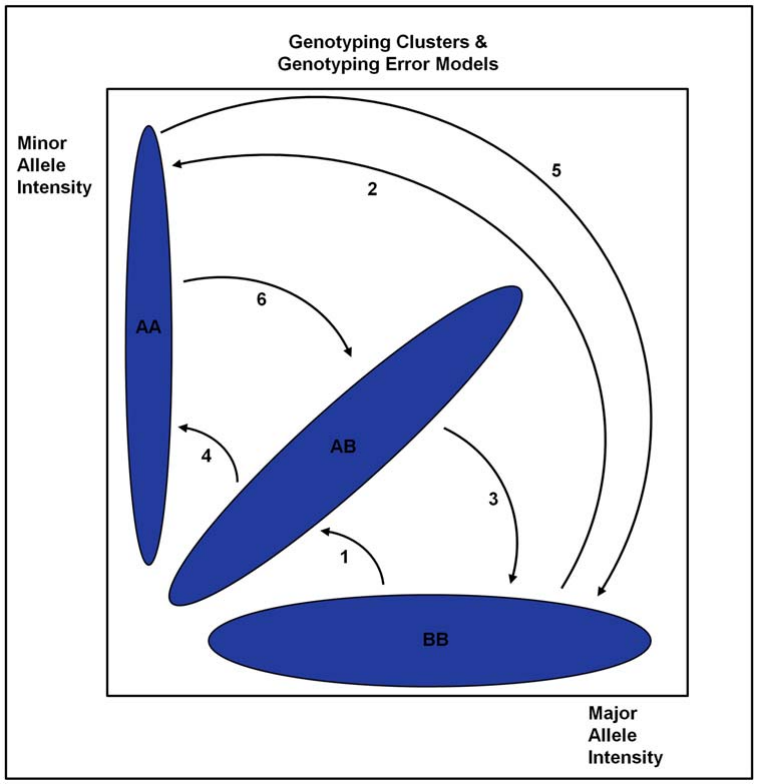
Genotyping error models. The three genotype clusters represent the clouds generated from intensity plots. The AA cluster consists of all homozygous minor calls, the AB cluster heterozygous calls and the BB homozygous major calls. Each arrow represents one of the genotyping error models considered. For example, in Model #6 minor homozygotes (AA) can be miscalled as heterozygotes (AB).

Using simulation studies, we examine the power of the transmission test statistic 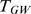 to detect and estimate the average genome-wide genotyping error rate for individual probands. Previous studies investigated genotyping error models that are specific to an individual SNP [18], [26]–[29]. In this communication, we examine genotyping error at a SNP-chip level where several thousand markers have been genotyped. The specification of a universal genotyping error model that is a reflection of the genotyping errors as they are encountered on a genome-wide SNP chip is not straightforward. Such a model depends on various, partly unknown, parameters, e.g. the true genotyping error rate, its dependence on the allele frequency, the DNA quality, chip quality, the selected genotyping platform, etc. We therefore assess the effect of all possible misclassifications for a particular genotype in separate simulations. For all possible combinations of miscalling genotypes (Figure 1), we estimate the average of the genome-wide transmission test statistic 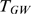. For each genotyping error/misclassification model, the average value for 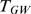 can then be used as an upper bound for assessing the average genotyping error rate within an individual proband.

In each replicate of the simulation study, we simulate data on 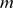 SNPs in one trio. The minor allele frequencies 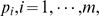 for each of the 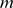 SNPs are randomly drawn either from a uniform (0.1,0.5) distribution or a beta (2,8) distribution (truncated so that 10%<MAF<50%), resembling SNP chips with evenly distributed allele frequencies and SNP chips with higher proportions of rare SNPs. Assuming Hardy-Weinberg equilibrium, the parental genotypes, 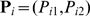, are generated by drawing twice from a binomial distribution with probability 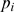, once for each parent. Then, using Mendelian transmission from the parents, proband genotypes are simulated. In order to understand the severity/effect of miscalling each genotype and its impact on the transmission test statistic 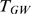, all possible genotyping error models of Figure 1 are considered separately in the simulation study. We assume that the probability of misclassifying one genotype as another genotype is denoted by 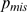, and errors are randomly generated in all three family-members, based on the genotyping error model (Figure 1). That is, each genotype is miscalled with probability, 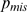. This process is repeated until genetic data for 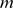 markers is created for the trio.

**Figure 2:**
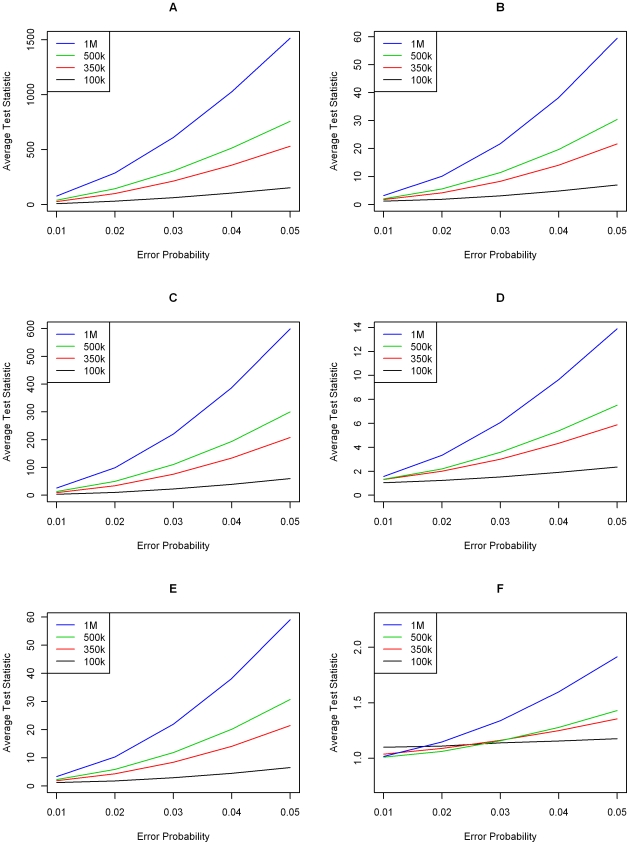
Simulation results—minor allele frequencies drawn from a truncated Beta (2,8) distribution. Average standardized transmission test over 1,000 replications for varying levels of genotype error and SNP chip sizes. Each graph displays results for a single genotype error model from Figure 1. (A–F) correspond to Models 1–6, respectively. Legends are different in each graph.

**Figure 3:**
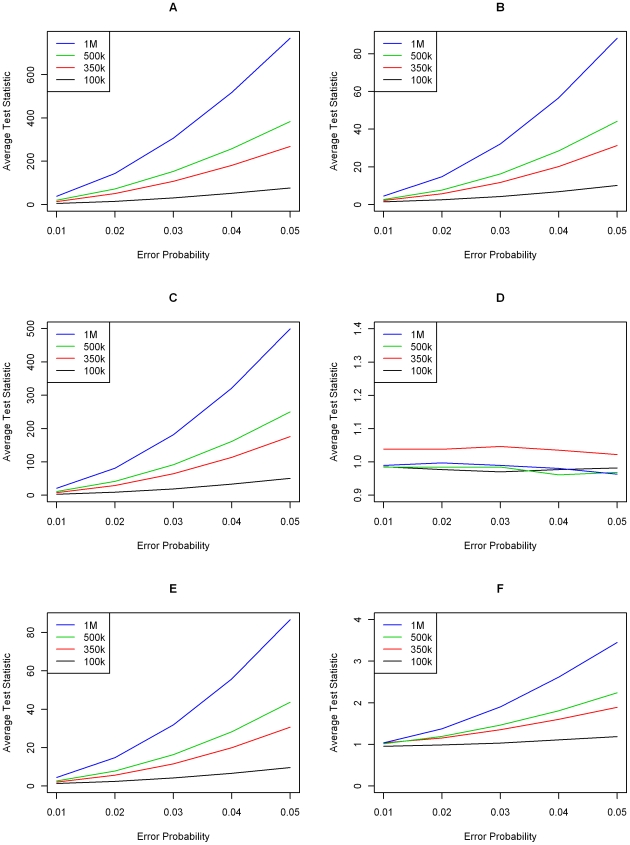
Simulation results—minor allele frequencies drawn from a Uniform (0.1,0.5) distribution. Average standardized transmission test over 1,000 replications for varying levels of genotype error and SNP chip sizes. Each graph displays results for a single genotype error model from Figure 1. (A–F) correspond to Models 1–6, respectively. Legends are different in each graph.

Quality control filtering is applied in order to identify trios with particularly bad quality data. Specifically, trios with Mendelian inconsistencies are removed. Then the standardized genome-wide statistic 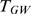 is calculated using all markers passing the quality control filtering. The average value of the test statistics over 1000 replications from both the beta and the uniform distributions are displayed below (Figure 2 and Figure 3, respectively). These reveal that the genome-wide transmission test statistic 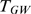 can show large deviations from the null hypothesis when the genotyping error rate is small to moderate. The transmission test statistic can therefore serve as a measure of the previously undetectable genotyping error within a single proband.

Investigating differences specific to genotyping error models, we see that the most severe deviations occur under Models 1 & 3, which involve miscalling of homozygous major genotypes as heterozygotes and vice versa. For example, under Model 1, a misclassification probability (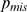) of 2%, a chip size of 350,000 markers and alleles generated from a beta distribution, the average value of the transmission test statistic was 101.12 and was 50.49 when the marker allele frequencies were drawn from a uniform distribution. This observation makes sense intuitively as these genotype classes are the most common. Since genotyping errors are less likely to be identified for heterozygous parents, this effect is further amplified. Miscalling between homozygous genotype clusters (Models 2 & 5) results in the next largest average test statistics followed by the models where heterozygotes and minor homozygotes are misclassified (Models 4 & 6).

Results between the two types of SNP chip, distinguished by the generation of minor allele frequencies, are relatively minor, with the average transmission test generally being higher using a beta distribution. Under either setting, probands can be identified with sufficient power when exhibiting genotyping error rates consistent with Models 1, 2, 3 & 5. That is, unless genotyping errors only come about by miscalling between heterozygote and minor homozygote genotype classes, the new transmission test statistic is powerful to detect probands who have remained unfiltered by traditional quality control measures.

### Linkage Disequilibrium

In the previous simulations, we assume the absence of linkage disequilibrium between the loci. However, the SNP density on most modern SNP chips is so high that the genotyped SNPs are in linkage disequilibrium. We therefore repeated the simulation experiments under the assumption that the analyzed SNPs are correlated. In the presence of LD, the minor allele frequency is randomly drawn from either a beta or a uniform distribution and is then adjusted for the presence of LD using the linkage disequilibrium parameter D and the minor allele frequency at the previously generated locus, i.e. 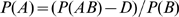, where A denotes the minor allele at the current locus and B the minor allele at the previously generated locus. The parameter D can be defined through the parameter 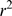 and be generated by drawing from a uniform distribution.

**Figure 4:**
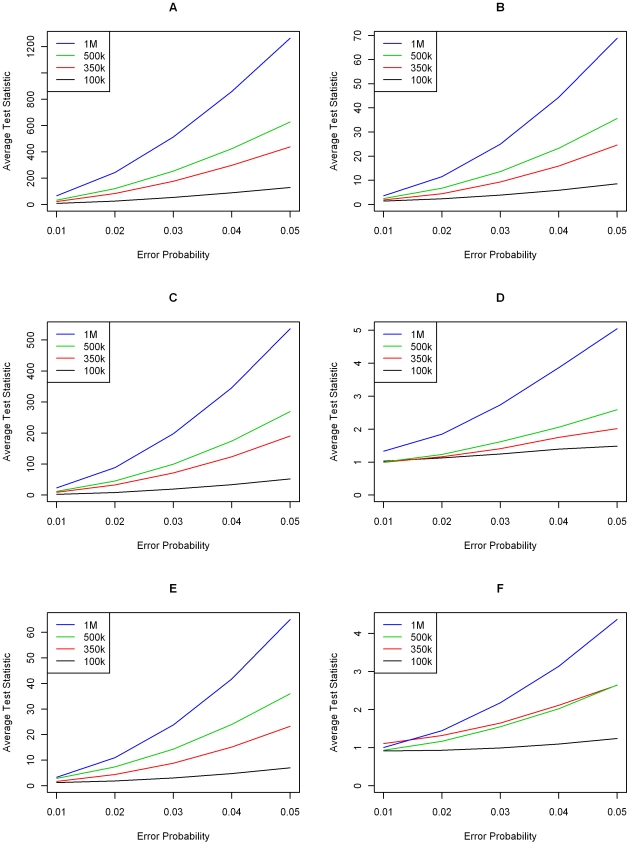
Simulation results with LD—minor allele frequencies drawn from a truncated Beta (2,8) distribution. Average standardized transmission test over 1,000 replications for varying levels of genotype error and SNP chip sizes in the presence of LD. Each graph displays results for a single genotype error model from Figure 1. (A–F) correspond to Models 1–6, respectively. Legends are different in each graph.

**Figure 5:**
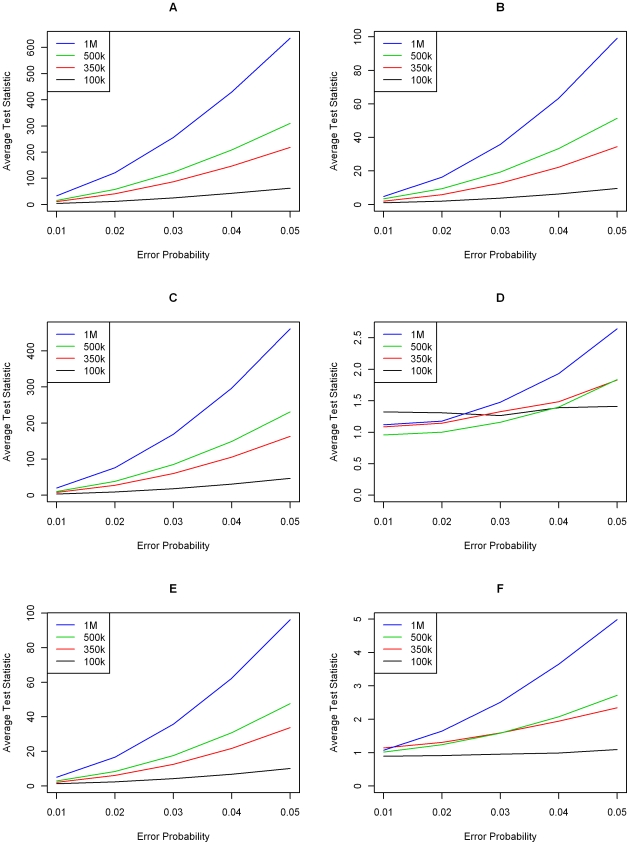
Simulation results with LD—minor allele frequencies drawn from a Uniform (0.1,0.5) distribution. Average standardized transmission test over 1,000 replications for varying levels of genotype error and SNP chip sizes in the presence of LD. Each graph displays results for a single genotype error model from Figure 1. (A–F) correspond to Models 1–6, respectively. Legends are different in each graph.

Figure 4 and Figure 5 display the average values of the standardized genome-wide test statistic over 1000 replicates under the previously considered scenarios with minor allele frequencies being generated as before, from a beta distribution and a uniform distribution, respectively. With the exception of genotyping error Models 2 and 5, the presence of LD between the SNPs leads to a small reduction in power of the genome-wide transmission statistic 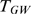. However, the power of the approach remains sufficiently large to identify probands with even small genotyping error rates. For the genotyping error Models 2 and 5, the presence of LD slightly increases the power of the genome-wide transmission test statistic 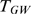. It is important to note that these two genotyping error models are extreme and probably not very realistic in the sense that the common homozygous genotype is misclassified as the rare homozygous genotype (Model 2) and vice versa (Model 5).

### Simulation Studies—Type I Error

To verify the theoretically derived distribution of 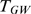 under the null hypothesis of no genotyping error, we conducted simulation studies mirroring all of the scenarios examined previously. The simulation studies were run in the absence and in the presence of LD. For each scenario, 10,000 datasets were generated and the empirical significance level was estimated by the proportion of replicates for which the genome-wide transmission test statistic 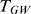 was significant at an 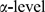 of 5%. Table 1 and Table 2 show the estimated significance levels in the presence and absence of LD. In all settings, the nominal Type I error rate of 5% is maintained by the test statistic.

**Table 1:** Empirical significance – Percentage of genome-wide transmission test false positives in 10,000 datasets with no LD.

Error Model	Uniform MAF				Beta MAF			
	100k	350k	500k	1M	100k	350k	500k	1M
1	0.050	0.049	0.050	0.049	0.049	0.049	0.047	0.047
2	0.051	0.050	0.048	0.049	0.053	0.045	0.048	0.049
3	0.050	0.049	0.047	0.052	0.051	0.050	0.048	0.050
4	0.046	0.045	0.051	0.051	0.049	0.051	0.051	0.055
5	0.049	0.049	0.053	0.051	0.048	0.048	0.049	0.048
6	0.051	0.054	0.050	0.050	0.050	0.049	0.048	0.045

Proportion of 10,000 datasets simulated under the null hypothesis of no genotyping error and without LD such that 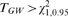. Columns 2 through 5 display results for various chip sizes when generating minor allele frequencies from a Uniform (0.1,0.5) distribution. Columns 6 through 9 display analogous results when generating minor allele frequencies from a Beta (2,8) distribution. Each row depicts results corresponding to a distinct genotyping error model from Figure 1.

**Table 2:** Empirical significance — Percentage of genome-wide transmission test false positives in 10,000 datasets with LD.

Error Model	Uniform MAF				Beta MAF			
	100k	350k	500k	1M	100k	350k	500k	1M
1	0.054	0.048	0.052	0.049	0.051	0.052	0.052	0.054
2	0.052	0.051	0.050	0.048	0.048	0.050	0.048	0.047
3	0.050	0.050	0.045	0.051	0.048	0.052	0.050	0.051
4	0.048	0.044	0.051	0.050	0.051	0.050	0.048	0.051
5	0.050	0.050	0.049	0.051	0.053	0.049	0.047	0.052
6	0.044	0.052	0.050	0.050	0.051	0.052	0.052	0.053

Proportion of 10,000 datasets simulated under the null hypothesis of no genotyping error and in the presence of LD such that 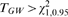. Columns 2 through 5 display results for various chip sizes when generating minor allele frequencies from a Uniform (0.1,0.5) distribution. Columns 6 through 9 display analogous results when generating minor allele frequencies from a Beta (2,8) distribution. Each row depicts results corresponding to a distinct genotyping error model from Figure 1.

### Application to HapMap data

To illustrate the practical relevance of the proposed genome-wide test for single probands, we applied the methodology to the third release of the HapMap data [30]. We analyzed 41 available probands in the CEPH (Utah residents with ancestry from northern and western Europe) family pedigrees with both parents gentoyped. The SNPs were generated by genotyping all probands with both the Affymetrix 6.0 chip and the Illumina 1M chip, providing total data on 1,403,896 SNPs.

The genotyping data was extensively cleaned as described at http://www.broad.mit.edu/~debakker/p3.html. For example, SNPs were filtered if, within a population, the Hardy-Weinberg test p-value was less than 10^−6^, missingness was greater than 5% or if there were three or more Mendelian errors.

For the analysis, we selected a cutoff of 5% for the minor allele frequency as a quality control filter. Each proband was analyzed three times. First, the genome-wide transmission test statistic was computed for all 1,403,896 available SNPs. The second analysis was focused on the 249,889 SNPs that were available on both genotyping platforms and provided concordant genotype calls for both SNP chips. For the third analysis the SNPs that were available on only one of the SNP chips but not on the other were examined. Table 3 shows the results of all 3 analyses for 41 CEPH probands.

**Table 3:** Genome-wide transmission test statistic for 41 CEPH probands.

Pedigree ID	Proband ID	All SNPs Analysis	Concordant SNPs Analysis	Single Platform Analysis
1330	12335	1.77	0.05	2.50
1330	12336	24.07	8.26	16.57
1334	10846	17.30	8.99	10.16
1334	10847	14.16	7.93	8.05
1340	7029	92.13	2.18	98.12
1341	6991	3.29	0.14	4.73
1345	7348	15.12	3.91	11.33
1345	7349	3.98	0.04	4.47
1347	10859	39.99	4.79	35.50
1350	10855	66.04	8.57	57.79
1350	10856	0.16	0.66	0.00
1353	12376	62.68	5.52	58.42
1354	12386	3.26	0.65	2.61
1362	10860	90.93	1.60	98.69
1362	10861	0.24	0.21	0.57
1375	10863	49.55	6.73	43.02
1377	10864	12.94	0.53	13.19
1408	10831	32.93	4.90	28.09
1416	10835	21.91	1.67	20.83
1418	10836	92.60	0.12	116.45
1418	10837	15.91	1.29	15.00
1420	10839	1.11	0.92	0.51
1421	10840	22.72	5.80	17.10
1423	10843	13.33	1.38	12.13
1424	10845	7.69	0.25	8.01
1444	12740	5.54	0.00	6.79
1447	12752	33.59	4.98	28.67
1447	12753	24.07	5.21	18.92
1451	12766	2.20	0.11	3.21
1451	12767	55.85	**16.08**	40.64
1454	12801	34.50	0.67	47.20
1454	12802	37.64	9.67	28.28
1456	12817	0.81	0.08	0.74
1456	12818	47.65	8.75	38.91
1458	12832	99.97	**20.15**	79.89
1459	12864	2.15	1.28	1.19
1459	12865	34.20	4.51	29.85
1463	12878	39.54	**11.93**	28.38
13281	12344	25.49	6.60	19.11
13291	6995	0.03	0.03	0.07
13291	6997	0.11	0.17	0.32
	**Medians**	21.91	1.67	16.57

Significance threshold at an overall *α*-level of 5% for *χ* ^2^-statistics adjusted for 41 comparisons using Bonferroni-correction: 10.46.

The genome-wide transmission test statistic, 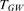, is reported for each CEPH proband with both parents genotyped, ordered by Pedigree ID. Each statistic is calculated using all available SNPs (Column 3), all concordant SNPs (Column 4) and the SNPs appearing on only one platform (Column 5). Test statistics using all concordant SNPs that are larger than the Bonferroni-adjusted value of 10.46 are presented in bold.

Given the additional quality control checking based on concordant genotype calls on both SNP chips, the second analysis will be based on the SNPs with very highest genotyping quality, while SNPs that are used in the third analysis are of considerably lower genotyping quality.

In the first analysis it is important to note that, for some probands, genome-wide transmission test statistics are observed that exceed values of 30, indicating substantial amounts of genotyping error in the data. The second analysis, which is focused on the SNPs that were available on both SNP chips and provided consistent genotyping results, produced much smaller test statistics for nearly all of the probands, even those who had very high transmission test results in the first analysis. Although 3 probands still show significant test results for the presence of genotyping error at an overall 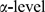 of 5%, adjusted for 41 comparisons using Bonferroni-correction, the actually observed values for the test statistic 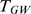 indicate that the genotyping error rate can be expected to be low.

In general for the second analysis, based on our previous simulations, we do not observe any probands that seem to have excessive amount of genotyping error. This observation is intuitively expected, since the second analysis is based only on genotype data that was concordant on both platforms and, therefore, should be of relatively high quality. In the third analysis, we again observe probands for whom the genome-wide transmission test indicates substantial amount of genotyping error. These probands are the same ones who also exhibited the high test statistic values in the first analysis. This result is expected as well since the third analysis is focused on the SNPs where genotype calls could not be confirmed by a second platform and are likely of poorer genotyping quality than the SNPs used in the second analysis.

As an exploratory analysis, we examined whether the probands with large values for the 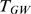 statistic could have been identified by other methods. If we had additionally applied filtering based on plotting each proband's mean heterozygosity versus the fraction of missing genotypes [31], only one proband would have been identified as an outlier. This proband is 1362: 10860 (Pedigree ID: Proband ID) and has 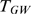 statistics of 90.93 when all genotyped SNPs are included in the analysis, 1.60 using the SNPs that are on both platforms and 98.69 for the analysis of SNPs that are only available on a single platform.

## Discussion

In this manuscript, we proposed a novel transmission test for the detection of genotyping errors in a single proband. In contrast to previously proposed family-based association tests, our approach can be applied just to a single proband with an arbitrary number of genotyped SNPs without the need to specify any LD structure. Our simulation results suggest that the genome-wide transmission test is sufficiently powerful to detect single probands with poor genotyping quality. This feature will allow the researcher to remove such probands from the dataset before the association analysis. Because the family-based association test statistic will be inflated regardless of which family member contains the genotyping error, we recommend removal of the entire nuclear family. In an application to the third release of the HapMap data, the proposed test was able to identify single probands with high genotyping error rates which are attributable to SNPs that could not be genotyped on both SNP chips. The key properties of the genome-wide test statistic, application to an arbitrary number of SNPs and an unspecified LD structure, will make the approach a useful tool for the quality control filtering in genome-wide association studies.
